# Anatomical and functional imaging of congenital heart disease with phase contrast VIPR

**DOI:** 10.1186/1532-429X-11-S1-P212

**Published:** 2009-01-28

**Authors:** Christopher J Francois, Elizabeth Nett, Kevin M Johnson, Darren Lum, Scott B Reeder, Oliver Wieben

**Affiliations:** University of Wisconsin, Madison, WI USA

**Keywords:** Congenital Heart Disease, Respiratory Gating, Scimitar Syndrome, CEMRA Image, Isotropic Projection

## Purpose

Demonstrate feasibility of PC VIPR for comprehensive cardiovascular imaging in congenital heart disease.

## Introduction

Imaging of congenital heart disease usually requires multiple anatomical and functional scans followed by several 2D phase contrast (PC) acquisitions in a variety of oblique scan planes for flow measurements. A new acquisition technique, PC vastly undersampled isotropic projection reconstruction (PC VIPR), that allows for volumetric 3D CINE flow imaging with high spatial and temporal resolution over a large field of view in a reasonable scan time has been developed [1]. The applicability of this approach has been extended by implementing a respiratory gating scheme that allows for free breathing acquisition [2]. The high spatial resolution with isotropic voxels improves image quality compared to standard PC MR by minimizing intravoxel dephasing effects and allows for retrospective data reformatting including flow measurements in arbitrary planes.

## Methods

A dual-echo PC VIPR acquisition with a PILS reconstruction and respiratory gating was implemented on our 1.5 T and 3 T clinical systems (GE Healthcare). To reliably achieve high quality images, several correction schemes were applied to account for the effects of T1-saturation, trajectory errors, motion, and aliasing associated with undersampling. Typical scan parameters were: 62.5 kHz receiver bandwidth, (1.0–1.25 mm^3^) isotropic spatial resolution in approximately 10 min scan time with 50% respiratory gating efficiency, imaging volume: 32 × 32 × 16 cm^3^, VENC of 50–100 cm/s (application specific). Cardiac gating was performed retrospectively with a temporal filter for radial acquisitions, similar to view sharing in Fourier sampling [3]. PC VIPR data were acquired after obtaining patient consent according to our IRB protocol in a total of 17 consecutive patients with a variety of pathology including aortic coarctation, Scimitar syndrome, double inlet left ventricle, and atrial septal defects, among others. The PC VIPR data were reconstructed as magnitude images, velocity vector fields, and angiograms calculated similar to complex difference images. CEMRA and 2D PC images were used for comparisons when available.

## Results

**Figure 1:**
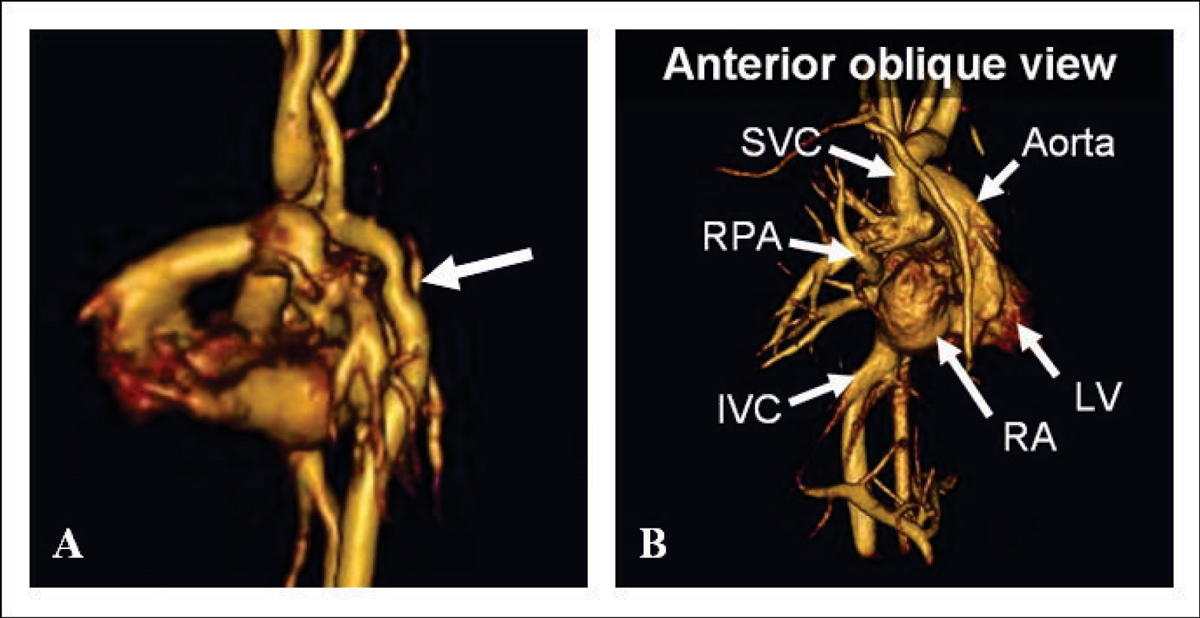
**Volume rendered images in (A) 2 month-old with aortic coarctation** ***(arrow)*** **and (B) patient with bidirectional Glenn for DILV**.

**Figure 2:**
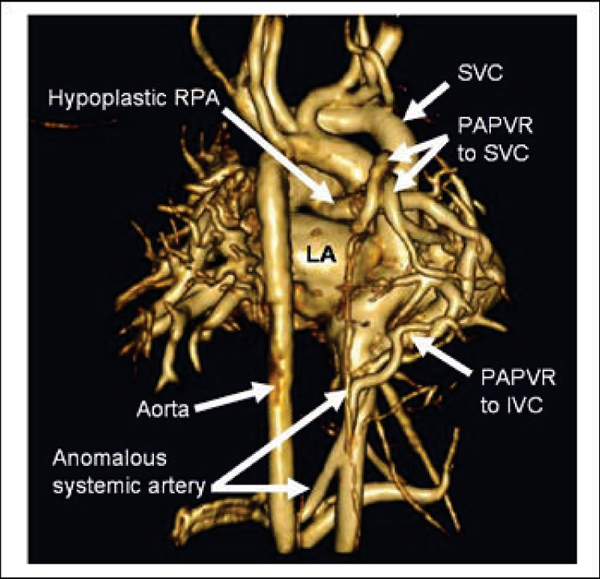
**Volume rendered image in 18 month-old with pulmonary venolobar syndrome consisting of hypoplastic right pulmonary artery (RPA), partial anomalous pulmonary venous return (PAPVR) to superior vena cava (SVC) and inferior vena cava (IVC), and anomalous systemic pulmonary artery from the abdominal aorta to the right lower lobe**.

**Figure 3:**
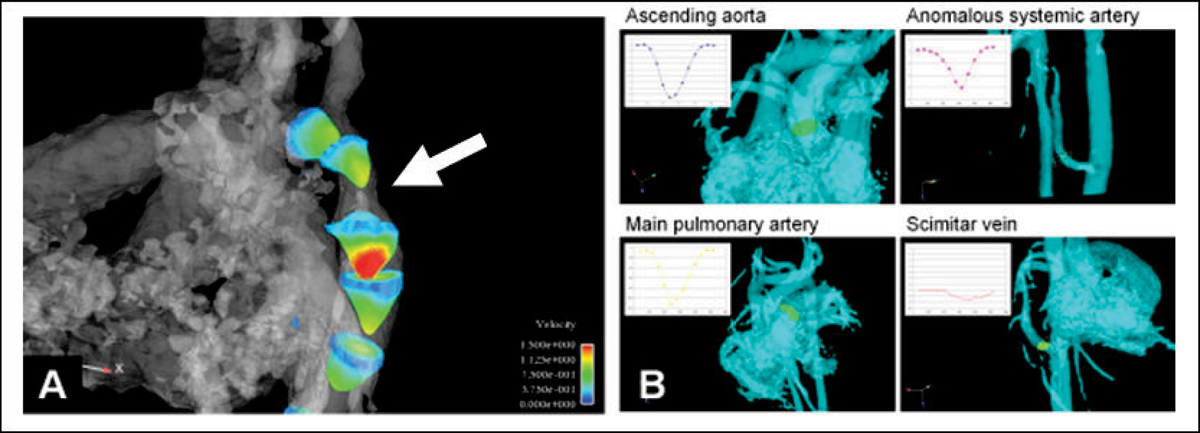
**(A) Flow velocity profiles in patient shown in Fig. 1a**. The highest velocity is immediately distal to coarcation (arrow). **(B)** Flow-time curves were measured at four different locations in patient shown in Fig. 2 using advanced visualization software. With PC VIPR, measurements can be made in any arbirtrary location after the images have been acquired.

PC VIPR data sets were successfully acquired in all patients. MR angiograms were created using the average flow PC VIPR dataset (Figure 1) to visualize cardiovascular structures. All anatomical structures visualized on CEMRA images were identified on PCVIPR images. This includes a 2 year-old with pulmonary venolobar syndrome with an anomalous pulmonary venous return to the SVC and IVC, in addition to an anomalous systemic artery (Figure 2). In addition, individual cardiac frames were reconstructed (with reduced SNR) and the velocity fields were visualized and analyzed with advanced software (EnSight), allowing for assessment of hemodynamics (Figure 3).

## Conclusion

Comprehensive anatomical and functional cardiovascular MRI of congenital heart disease can be performed using PC VIPR, providing a powerful new tool for noninvasive diagnosis.
